# The rupture mechanism of rubredoxin is more complex than previously thought†

**DOI:** 10.1039/d0sc02164d

**Published:** 2020-05-27

**Authors:** Maximilian Scheurer, Andreas Dreuw, Martin Head-Gordon, Tim Stauch

**Affiliations:** Interdisciplinary Center for Scientific Computing Im Neuenheimer Feld 205 69120 Heidelberg Germany; Department of Chemistry, University of California Berkeley California 94720 USA; Chemical Sciences Division, Lawrence Berkeley National Laboratory, University of California Berkeley California 94720 USA; University of Bremen, Institute for Physical and Theoretical Chemistry Leobener Straße NW2 D-28359 Bremen Germany tstauch@uni-bremen.de; Bremen Center for Computational Materials Science, University of Bremen Am Fallturm 1 D-28359 Bremen Germany; MAPEX Center for Materials and Processes, University of Bremen Bibliothekstraße 1 D-28359 Bremen Germany

## Abstract

The surprisingly low rupture force and remarkable mechanical anisotropy of rubredoxin have been known for several years. Exploiting the first combination of steered molecular dynamics and the quantum chemical Judgement of Energy DIstribution (JEDI) analysis, the common belief that hydrogen bonds between neighboring amino acid backbones and the sulfur atoms of the central FeS_4_ unit in rubredoxin determine the low mechanical resistance of the protein is invalidated. The distribution of strain energy in the central part of rubredoxin is elucidated in real-time with unprecedented detail, giving important insights into the mechanical unfolding pathway of rubredoxin. While structural anisotropy as well as the contribution of angle bendings in the FeS_4_ unit have a significant influence on the mechanical properties of rubredoxin, these factors are insufficient to explain the experimentally observed low rupture force. Instead, the rupture mechanism of rubredoxin is far more complex than previously thought and requires more than just a hydrogen bond network.

## Abstract 2

Using steered molecular dynamics simulations and strain analysis it is shown that, in contrast to previous assumptions, the experimentally found low rupture force of the iron–sulfur-bond in rubredoxin cannot be explained by hydrogen bond networks.

## Graphical abstract

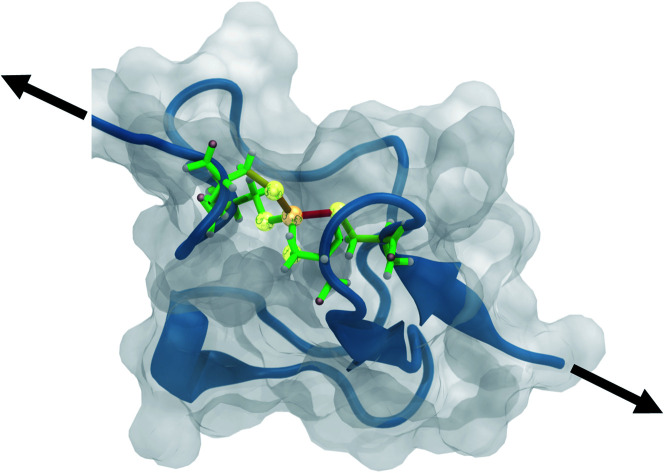

## 1 Introduction

Proteins are exposed to mechanical forces during their entire life spans, including their synthesis, folding and unfolding events, the execution of their diverse tasks in the cell, and ultimately their degradation.^1^ The remarkable elastic properties of muscles^2^ and the mechanical resilience of silk^3^ are only two examples where the response of proteins to forces leads to important macroscopic effects. Not surprisingly, this fascinating field of mechanobiology has been studied extensively within the past two decades or so, by using both experimental and computational methods,^4^ and it was found that proteins display a remarkably rich behavior when exposed to mechanical forces.^1^ In many cases, the response of proteins to stretching is unexpected or even counterintuitive: metalloproteins, for example, are often much weaker than one would expect from the covalent character of the involved metal–ligand bonds.^5,6^ A prime example for this effect is found in rubredoxin, a protein that participates in electron transfer reactions^7–9^ and provides structural stability due to its central FeS_4_ unit.^9^ Using atomic force microscopy (AFM), it was found that the strong covalent character of the iron–sulfur bonds^10^ of the protein's central FeS_4_ unit contrasts strikingly with a remarkably low rupture force of approx. 200 pN.^11^ In contrast, covalent bonds typically display rupture forces above 1.5 nN.^12^ Due to the central role of iron–sulfur clusters in biochemistry, the mechanical properties of rubredoxin were investigated diligently, using both experimental and computational approaches. It was found that the cleavage of the Fe–S bond in rubredoxin typically proceeds *via* a homolytic pathway,^13,14^ however, heterolytic bond rupture has been described if nucleophiles are present.^15^ Not surprisingly, the rupture force also depends crucially on the chemical species in the environment of the FeS_4_ unit in rubredoxin.^15^ Moreover, the mechanism and the kinetics of rupture have been shown to depend on the pulling direction.^16^ This mechanical anisotropy of rubredoxin has also been confirmed by steered molecular dynamics (SMD) simulations.^17^

**Fig. 1:**
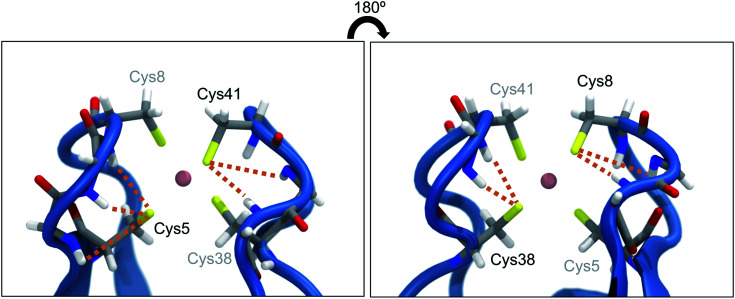
Close-up view of the central FeS_4_ unit in rubredoxin (PDB 1BRF). Dotted lines represent possible hydrogen bonds between the sulfur atoms and the closest amino acid backbones (Cys5: Lys6, Ile7, Cys8; Cys8: Gly9, Tyr10; Cys38: Ile40, Cys41; Cys41: Gly42, Ala43) according to ref. 18. The structure in the right panel is turned by 180° w.r.t. the structure in the left panel.

The presence of hydrogen bonds (NH⋯S) between neighboring amino acid backbones and the sulfur atoms of the FeS_4_ unit (Fig. 1) has been suggested as a possible explanation for the surprisingly low rupture force of the Fe–S bonds in rubredoxin^19^ by decreasing the covalent character of the Fe–S bonds. Similar effects have been previously identified in the context of a modulation of reduction potentials in rubredoxin by hydrogen bonds.^20^ Interestingly, however, rubredoxin model systems with intramolecular NH⋯S hydrogen bonds exhibit mean Fe–S bond lengths that are significantly shorter than those of comparable complexes that do not form hydrogen bonds.^21^ Considering that a short bond is typically interpreted as strong,^4^ this finding seems to be in conflict with the notion that NH⋯S hydrogen bonds weaken the Fe–S bonds in rubredoxin.

In this paper, SMD in combination with quantum chemical strain analysis elucidates the dynamic strain fluctuations during the mechanical unfolding of rubredoxin in unprecedented spatial and temporal resolution. Furthermore, density functional theory (DFT) is used to investigate the mechanical resilience and anisotropy of rubredoxin in detail. Hydrogen bonds between neighboring amino acid backbones and the sulfur atom of the central FeS_4_ pseudo-tetrahedron are shown to play only a minor role in the mechanical properties of rubredoxin. The protein's mechanical resistance is influenced by structural anisotropy and angle bendings in the FeS_4_ unit, as evidenced by state-of-the-art strain analyses. However, these effects are not sufficient to explain the experimentally observed low rupture force of rubredoxin, thus hinting at a rupture mechanism that is significantly more complex than previously thought.

## 2 Computational methods

### 2.1 Steered molecular dynamics combined with quantum chemical strain analysis

Atomic coordinates were obtained from a *Pyrococcus furiosus* rubredoxin crystal structure (PDB: 1BRF).^22^ Hydrogen atoms were added using the VMD^23^*psfgen* plugin, and the protein was placed inside a water box with 0.15 M NaCl. For all molecular dynamics (MD) simulations, the CHARMM36 force field^24^ was applied together with NAMD, version 2.13.^25^ The charges of Fe(iii) and cysteine residues of rubredoxin were re-parametrized as explained in the ESI.† The anharmonic Fe–S bond potential was modeled through a Morse potential with parameters taken from previously published work^17^ (*D*_e_ = 90 kJ mol^−1^, *β* = 30 nm^−1^, *r*_0_ = 2.3 Å). The system was first minimized and subsequently equilibrated at a temperature of 300 K with a time step of 2 fs for 1 ns in total. During equilibration, backbone atoms of the protein were harmonically constrained with a force constant of *k* = 1.0 kcal mol^−1^ Å^−2^. Afterwards, a longer equilibration simulation was carried out for 25 ns without any constraints. For further analysis of hydrogen bonds to cysteine residues in the active site, an extended equilibration simulation of 250 ns length was performed. Starting from the equilibrated protein structure, SMD simulations were carried out to unfold the rubredoxin protein. In these SMD simulations, the Cα atom of Ala1 was fixed in space, whereas the Cα atom of the C-terminal Asp53 was pulled away from Ala1 along the bond axis at a constant velocity of 0.2 nm ns^−1^ employing a force constant of *k* = 7.0 kcal mol^−1^ Å^−2^. Snapshots of the trajectory were saved every 20 ps and the SMD trajectories were run until at least the first Fe–S bond rupture occurred. Ten SMD runs were run in total, and for the strain analysis a single trajectory was considered in the following due to the enormous computational cost of the workflow. Hydrogen bond analysis in equilibrium and in all ten SMD trajectories was carried out using PyContact,^26^ taking into account distance criteria from ref. 18 (S–H distance ≤3.0 Å). Possible hydrogen bond donors are Lys6, Ile7, Cys8, Gly9, Tyr10, Ile40, Cys41, Gly42, and Ala43.^18^ Distance analyses were performed with MDAnalysis.^27,28^

**Fig. 2:**
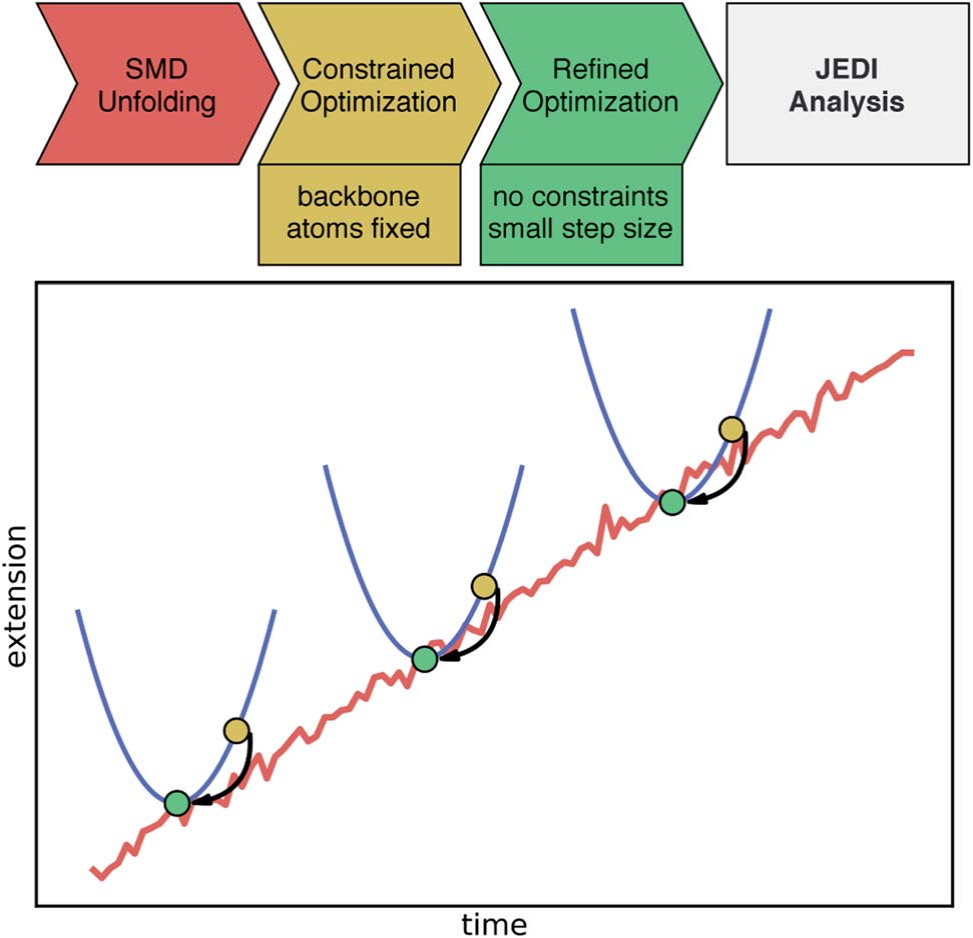
Schematic illustration of the applied workflow for the JEDI analysis along the SMD unfolding trajectory. The upper part shows the individual steps toward the JEDI analysis. The (non-quantitative) plot illustrates the SMD trajectory leading to a certain extension of the protein (red). Certain snapshots are extracted and quantum-chemically optimized keeping protein backbone atoms fixed (yellow dots). Then, the nearest local minimum, needed for the JEDI analysis, is found through a constraint-free optimization of the previous geometry (green dot). In this manner, pairs of strained and relaxed structures along the unfolding trajectory are obtained, yielding a well-defined JEDI analysis per snapshot.

To obtain an improved temporal resolution of the bond rupture event, the chosen SMD trajectory was restarted 1 ns before bond rupture while saving a snapshot every 0.1 ps. The quantum chemical Judgement of Energy DIstribution (JEDI) analysis was applied to investigate the partitioning of strain energy among all bonds, bendings and torsions of a mechanically strained molecule.^29–31^ Although force analyses by molecular dynamics simulations have been conducted for proteins,^32,33^ this is the first study to carry out the quantum chemical JEDI analysis along the unfolding trajectory of a protein. The workflow for this mixed quantum mechanical/molecular mechanics (QM/MM) approach is illustrated in Fig. 2. Snapshots including the four cysteine residues and Fe(iii) were extracted from the previous trajectory up to 9 ps before bond rupture. Broken bonds were saturated with hydrogen atoms. For each snapshot, a geometry optimization with density functional theory (DFT)^34,35^ at the BP86VWN^36,37^/6-31G(d)^38^ level of theory was performed while keeping the backbone atoms of the amino acids fixed to provide a strained geometry for each snapshot. The BP86VWN functional was chosen because it offers an attractive compromise between agreement with experimental data (see ESI, Fig. S1–S4†) and computational effort. Afterwards, a follow-up optimization without constraints was carried out with a tiny step size (Q-Chem keyword geom_opt_dmax = 20) to find the nearest local minimum, resulting in the relaxed reference geometry for that particular snapshot. Finally, JEDI analyses were carried out for each snapshot.

### 2.2 Static quantum chemical calculations

The minimal quantum chemical model systems under investigation consist of Fe(iii) or Fe(ii) surrounded by four SCH_3_^−^ residues in a pseudo-tetrahedral geometry, *i.e.* [Fe(iii)(SCH_3_)_4_]^−^ and [Fe(ii)(SCH_3_)_4_]^2−^, which have been used in previous computational studies.^13^ A high-spin ground state was assumed for the iron atom in all calculations. All calculations on the model systems were carried out at the BP86VWN^36,37^/def2-TZVP^39^ basis set. Forces were applied to a pair of carbon atoms in the methyl groups using the External Force is Explicitly Included (EFEI)^40–42^ approach, in which a constant external force is added to the nuclear gradient of a pair of atoms in each step of an otherwise relaxed geometry optimization. This additional force points outward along the connection line between the two atoms, driving them apart. The geometry optimization converges when the externally applied force and the internal restoring force of the molecule cancel. Rupture forces were determined iteratively with a resolution of 10 pN. All calculations were conducted using the Q-Chem 5.1 (ref. 43) program package.

The characterization of the bonding situation in the FeS_4_ cluster was achieved with the Localized Orbital Bonding Analysis (LOBA),^44^ in which Edmiston-Ruedenberg (ER) orbitals^45^ in combination with Löwdin population numbers^46^ are evaluated. ER orbitals minimize the non-classical interorbital exchange, thus leading to the most “classical” picture of bonding. LOBA has been used successfully to reveal the intricacies of bonding in various metal complexes, including such with a metal center surrounded by four sulfur atoms.^47^ Furthermore, the bonding situation was investigated *via* Energy Decomposition Analysis using Absolutely Localized Atomic Orbitals (ALMO–EDA),^48–51^ which are expanded in terms of the atomic orbitals localized on a specific fragment. The SCH_3_^−^ ligand that is ruptured by a sufficiently strong force was defined as one fragment and the rest of the complex was defined as the other fragment. Calculating the charge-transfer energy Δ*E*_CT_ in ALMO–EDA^52^ allows for an estimate of the covalent character of the Fe–S bond.

To obtain more detailed insights into the distribution of strain energy in the distorted model systems, the JEDI strain analysis was applied also in the static case. The strained geometries for this analysis were prepared according to a protocol described previously,^53^ in which the molecule is stretched until rupture is imminent, subsequently relaxed and then stretched again with different forces, whereby the problematic flipping of dihedral angles is avoided. All visualizations of molecular structures (strained and unstrained) were created with VMD.^23^

## 3 Results and discussion

### 3.1 Dynamics of the rubredoxin rupture process

**Fig. 3:**
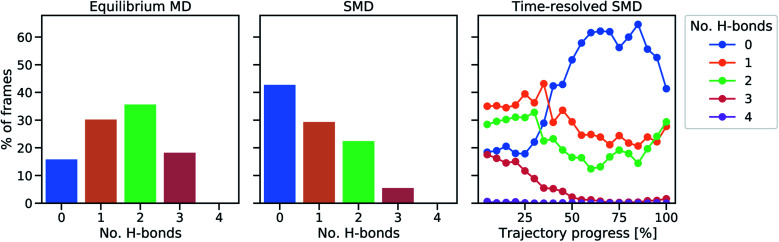
Analysis of protein hydrogen bonds to sulfur atoms of Cys5 and Cys41. The percentage of frames in which a certain number of hydrogen bonds occurs in equilibrium (left) and during the SMD unfolding procedure (middle). In the right panel, a time-resolved representation of hydrogen bond percentages is shown by averaging 20 equally sized trajectory windows. During the final parts of the trajectories, some of the Fe–S distances are already significantly elongated (*cf.* Fig. S5†).

In all SMD trajectories, one of the Fe–S bonds ruptures. In six of the ten trajectories, the sulfur atom involved in the bond rupture belongs to Cys5 and in the other four trajectories it belongs to Cys41. Hence, in a first step, the hydrogen bond network of the sulfur atoms belonging to Cys5 and Cys41 was analyzed in the equilibrium and in the steered unfolding trajectories (Fig. 3) to determine the influence of the hydrogen bond network on the mechanical properties of rubredoxin. In the equilibrium trajectory, most frames display either one or two hydrogen bonds in Cys5 and Cys41, with a minority of frames showing either zero or three hydrogen bonds. While it is of course possible that these hydrogen bonds influence the reactivity of the central FeS_4_ unit in the force-free state of rubredoxin, a similar analysis carried out during the unfolding trajectories prior to rupture of the Fe–S bond shows that the formation of NH⋯S hydrogen bonds becomes less and less frequent with increasing stress: in almost half of the frames during the unfolding trajectories, no such hydrogen bond is formed. The formation of one or two hydrogen bonds occurs less frequently and only a very small minority of frames throughout the unfolding events displays three hydrogen bonds. The prevalence of hydrogen bonds during the unfolding trajectory decreases almost continuously as rupture is approached (Fig. 3, right panel). As an exception, in the last 5–10% of the shown trajectories hydrogen bonds on average become more frequent again. At these points, the mean iron–sulfur distances have already increased significantly and approach the value of a ruptured bond (Fig. S5†). In agreement with chemical intuition, the sulfur atoms tend to form stabilizing hydrogen bonds if this is sterically feasible, *i.e.* if they are not screened by the rest of the FeS_4_ unit. Hence, up to the point of rupture of the iron–sulfur bond, the mean amount of hydrogen bonds to the sulfur atoms decreases. All in all, these observations demonstrate that hydrogen bonds to the sulfur atoms of the FeS_4_ unit are unlikely to determine the low rupture force of rubredoxin found in the experiments, since close to the bond rupture of the Fe–S bond these hydrogen bonds become less prevalent.

**Fig. 4:**
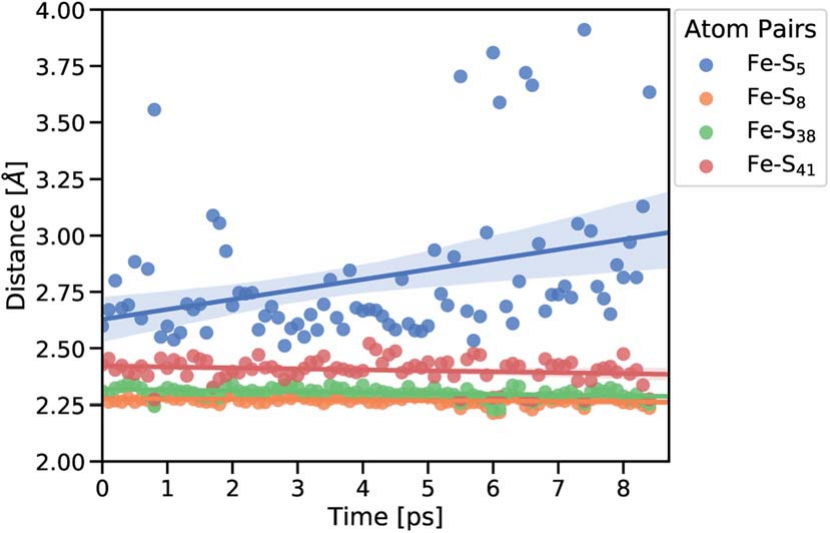
Fe–S distances of strained geometries before bond rupture. Subscripts indicate the amino acid residue number. Linear fits of the distances are displayed together with the confidence interval (translucent areas).

Subsequently, we focused on elucidating the real-time propagation of strain energy in the central part of rubredoxin during unfolding. To this end, we applied the workflow summarized in Fig. 2 to one of the SMD trajectories to derive strain energies using the JEDI analysis. As mentioned before, it is a stochastic process whether Cys5 or Cys41 ruptures. In the case of the particular unfolding trajectory considered here, the scissile bond is Fe–S_5_ (where “5” represents amino acid residue Cys5), as can be observed from the progression of the Fe–S distances in Fig. 4. Together with the Fe–S_41_ bond, which is also being elongated to a certain extent, the scissile Fe–S_5_ bond is part of the force-bearing scaffold of rubredoxin, because it lies along the connection line between the attachment point and the pulling point in the SMD simulation. The Fe–S_5_ bond distance oscillates tremendously during the trajectory, which is a result of the dynamic nature of the calculations, and partially breaks and reforms several times during the last 9 ps before it is broken completely. The remaining Fe–S bonds (Fe–S_8_ and Fe–S_38_), in turn, stay very close to their equilibrium bond lengths, since the force is acting almost perpendicular to them.

**Fig. 5:**
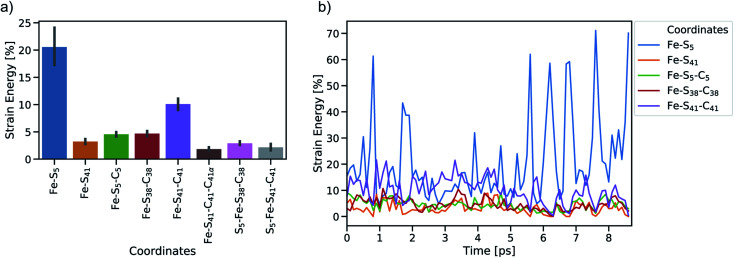
Summary of JEDI analyses including (a) mean strain energy percentages for the most strained coordinates (bond lengths Fe–S_5_ and Fe–S_41_, bond angles Fe–S_5_–C_5_, Fe–S_38_–C_38_ and Fe–S_41_–C_41_, and torsions Fe–S_41_–C_41_–C_41α_, S_5_–Fe–S_38_–C_38_ and S_5_–Fe–S_41_–C_41_) and (b) time-resolved strain energy contributions (percentages of the total strain) of the most important bond lengths and bendings. Bars around the mean percentages indicate the 95% confidence interval.

To study the dynamic propagation of mechanical strain energy during the unfolding process, the JEDI analysis was carried out at each snapshot. Considering the last 9 ps before the ultimate scission of the Fe–S_5_ bond, most strain energy is stored in the Fe–S_5_ bond itself (Fig. 5a). A significant amount of strain energy is also stored in the Fe–S_41_ bond as well as in several bond angles that lie along the connecting line of the attachment point and the pulling point of the SMD simulation. Together, these internal coordinates comprise the force-bearing scaffold of the central part of rubredoxin. Dihedral angle displacements play only a minor role in this trajectory. The significant role of the Fe–S_5_ bond and the Fe–S_41_–C_41_ bond angle as reservoirs of strain energy is further emphasized when considering the dynamic progression of strain energy (Fig. 5b). As expected, the amount of strain energy in the scissile Fe–S_5_ bond increases dramatically when approaching the point of bond rupture.

**Fig. 6:**
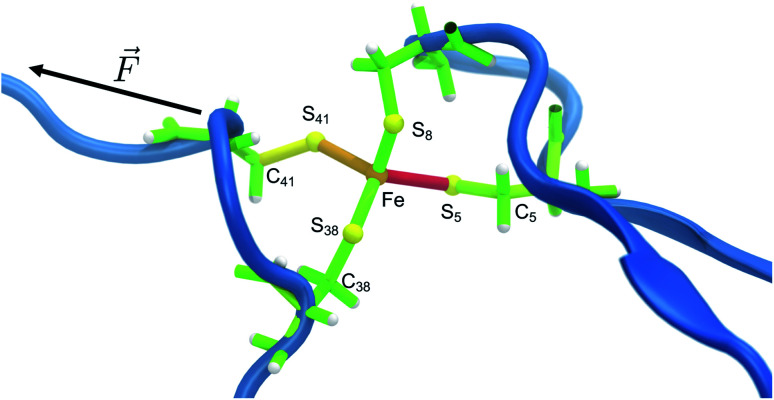
Representative snapshot of the unfolding trajectory before bond rupture. The strain energy contributions in the quantum region are mapped onto the bonds, where red and green indicate high and low strain, respectively, and transitions are fluent. The residues along the force coordinate bear most of the strain energy (Cys5 and Cys41), whereas Cys8 and Cys38 only play a minor role. The scissile bond Fe–S_5_ contains most of the strain energy, followed by the angle on the opposite side (Fe–S_41_–C_41_).

The distribution of strain energy in a representative snapshot is summarized by using a color-coded representation in Fig. 6. The force-bearing scaffold can be clearly distinguished: as expected, it lies along the connecting line between the attachment point and the pulling point. In the SMD trajectory, most strain energy is stored in the scissile Fe–S_5_ bond, but the other side of the FeS_4_ pseudo-tetrahedron is also strained significantly.

### 3.2 Modeling the rupture process in detail

To obtain more detailed insights into the underlying mechanism of rubredoxin scission, the model systems [Fe(iii)(SCH_3_)_4_]^−^ and [Fe(ii)(SCH_3_)_4_]^2−^ were investigated using static quantum chemical methods. For these model systems, the calculated rupture forces amount to 1.89 nN ([Fe(iii)(SCH_3_)_4_]^−^) and 1.01 nN ([Fe(ii)(SCH_3_)_4_]^2−^), respectively, which is significantly higher than the experimentally observed values in rubredoxin (258 ± 122 pN for the Fe(iii) system and 152 ± 62 pN for the Fe(ii) system). While calculated rupture forces are typically higher than experimental ones,^4,12,54^ the observed overestimation of the rupture force by static quantum chemical calculations demonstrates that the minimal model systems do not mimic the experimental conditions realistically. However, it is interesting to note that the calculated rupture force of [Fe(iii)(SCH_3_)_4_]^−^ is very similar to the rupture force of the weak transannular carbon–carbon bond in Dewar benzene,^55^ hinting towards a covalent character of the Fe(iii)–S bond. Further evidence is provided by a Localized Orbital Bonding Analysis (LOBA, *cf.* Fig. S6 and S7†),^44^ which demonstrates a higher covalent character of the Fe(iii)–S bond compared to the Fe(ii)–S bond. Intuitively, Fe(iii) attracts the electron density of the negatively charged SCH_3_^−^ ligands much more than Fe(ii), leading to a bond in which the electron density is shared more uniformly between the iron and sulfur atoms. Hence, the covalent character of this bond is significant, leading to a relatively high rupture force.

**Fig. 7:**
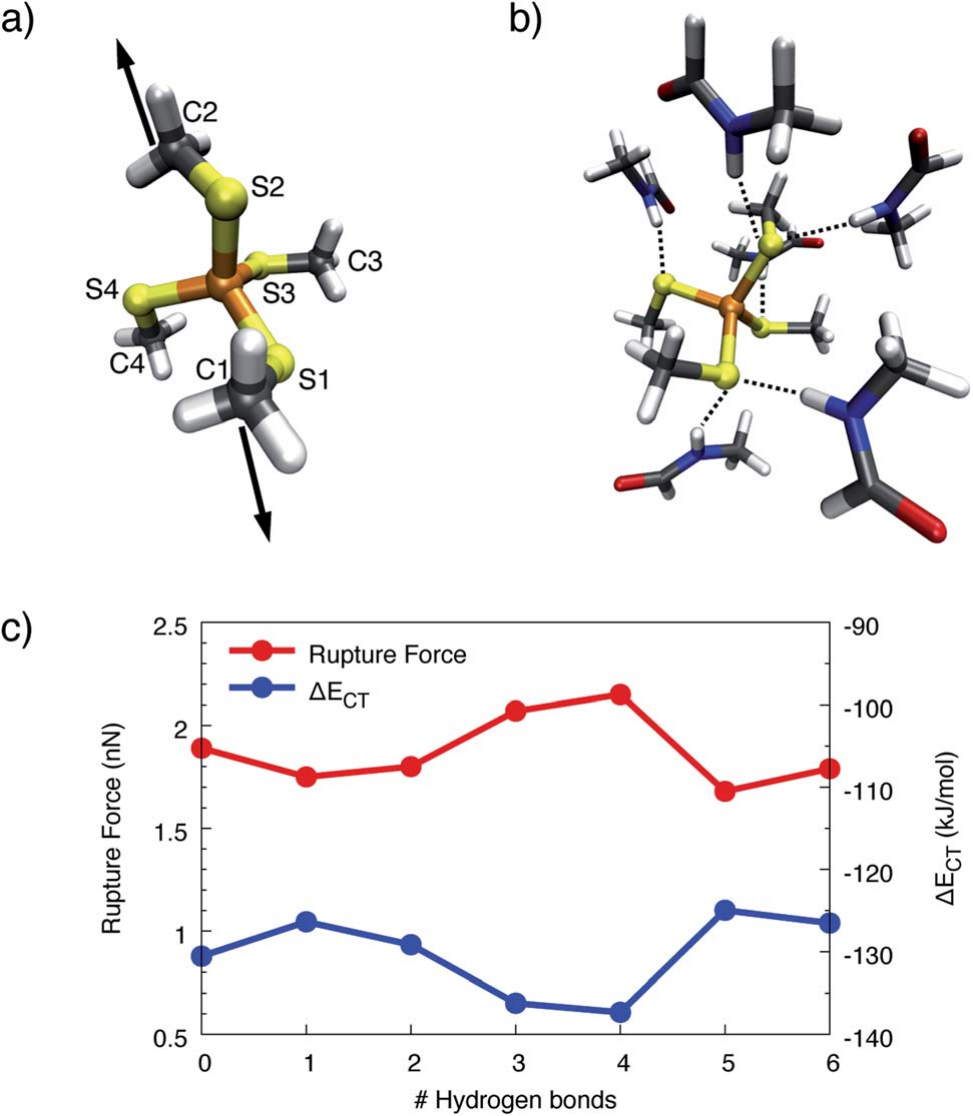
(a) Simplified numbering scheme used in the quantum chemical model system. Stretching forces were applied to the carbon atoms C1 and C2. (b) Quantum chemical model system with the complete set of formamide molecules forming hydrogen bonds (dotted lines) to the sulfur atoms of the FeS_4_ unit. (c) Rupture forces and charge-transfer energies Δ*E*_CT_ calculated for the model system [Fe(iii)(SCH_3_)_4_]^−^ with a varying number of hydrogen bonds formed between formamide molecules and the sulfur atoms. Lines were included to guide the eye.

In the SMD unfolding trajectories it was found that hydrogen bonds to the sulfur atoms of the central FeS_4_ unit become significantly less prevalent, thus leading us to the conclusion that such hydrogen bonds are unlikely to determine the mechanochemistry of rubredoxin. To corroborate the limited role of possible hydrogen bonds in more detail, an increasing number of formamide molecules forming hydrogen bonds to the sulfur atoms similar to those present in rubredoxin was added in the static quantum chemical calculations. The formamide molecules serve as models for the protein backbone of rubredoxin and were placed next to the sulfur atoms in the order S1, S2, S3, S4, S1, and S2 (*cf.*Fig. 7a for the numbering scheme), since these six hydrogen bonds were discussed in the structure of rubredoxin^19^ and were found in our SMD simulations. Subsequently, the carbon atoms C1 and C2 were pulled apart and the rupture forces for the [Fe(iii)(SCH_3_)_4_]^−^(HCONH_2_)_*n*_(*n* = 0–6) model complexes were determined (Fig. 7c).

Surprisingly, adding one or two formamide molecules to the sulfur atoms S1 and S2, which are attached to the carbon atoms that are pulled apart, leads to only a very minor decrease in rupture forces of less than 10%. Even more surprisingly, adding hydrogen bonds on the other side of the molecule, *i.e.* at the seemingly innocent sulfur atoms S3 and S4 in the pulling event (“3” and “4” on the *x*-axis in Fig. 7c), increases the rupture force to a value that lies significantly above the rupture force of the bare [Fe(iii)(SCH_3_)_4_]^−^. Hence, the change in electronic environment in the vicinity of the iron atom has a profound influence on the rupture force. Adding a second formamide molecule to the sulfur atoms S1 and S2 (“5” and “6” on the *x*-axis in Fig. 7c), again leads to a decrease in rupture force to a value slightly lower than the rupture force of the bare complex. Analogous results were obtained for [Fe(ii)(SCH_3_)_4_]^2−^ (Fig. S8†).

Although a hydrogen bond at a given sulfur atom can increase the corresponding Fe–S bond length by up to 0.029 Å (Fig. S9†), the covalent character of the Fe–S bond does not decrease if the sulfur atom is involved in hydrogen bonds. This conclusion can be drawn from the progression of the charge-transfer part of the bonding energy, Δ*E*_CT_, calculated *via* ALMO–EDA, when formamide molecules are added (Fig. 7c). The curve of Δ*E*_CT_ for [Fe(iii)(SCH_3_)_4_]^−^ is almost exactly a mirror image of the curve of rupture forces, demonstrating a higher amount of inter-fragment stabilization due to charge-transfer interaction to lead to a stronger covalent character and hence a larger rupture force. Although the rupture forces calculated with static quantum chemical methods overestimate the experimental values tremendously, the results from the static calculations thus support the observation that the presence of hydrogen bonds alone does not suffice to explain the surprisingly low rupture force observed in the experiment.

**Fig. 8:**
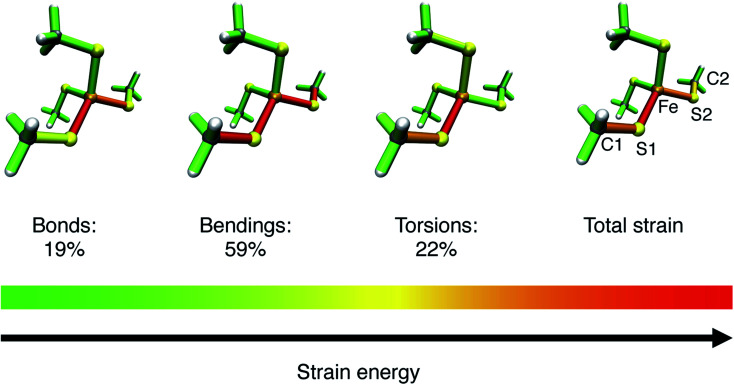
Color-coded distribution of harmonic strain energy among the bonds, bendings and torsions of [Fe(iii)(SCH_3_)_4_]^−^ at a stretching force of 1 nN, as calculated with the JEDI analysis. The picture for the total strain includes the specification of the numbering scheme used to identify the strained bonds and bendings, which is a subset of the scheme specified in Fig. 7.

To gain more detailed insights into the distribution of strain energy in bare [Fe(iii)(SCH_3_)_4_]^−^, the Judgement of Energy DIstribution (JEDI)^29–31^ analysis was employed. The progression of strain energy with increasing stretching forces can be found in the ESI (Fig. S11†). As an example, the distribution of strain energy among the bonds, bendings and torsions of [Fe(iii)(SCH_3_)_4_]^−^ at a constant stretching force of 1 nN driving the atoms C1 and C2 apart is given in Fig. 8. The force-bearing scaffold, involving all internal coordinates on the connecting line between C1 and C2, can be identified clearly. However, the distribution of strain energy is anisotropic: The bond Fe–S1 stores more strain energy than the chemically equivalent bond Fe–S2, preconditioning the former for rupture, and a certain amount of strain dissipates into the seemingly uninvolved part of the pseudo-tetrahedron *via* torsions. Moreover, the strain energy in the bendings is significantly higher than in the bonds. Hence, the FeS_4_ pseudo-tetrahedron in rubredoxin is a prime example of a system in which the deformation of internal coordinates other than the stretching of the scissile bond determine the mechanochemistry, which has been observed previously for bendings^56^ and torsions.^53^ The importance of bond angle bendings in the rupture process is highlighted by the observation that constraining the terminal bond angles yields a higher rupture force than an unconstrained EFEI calculation: it increases from 1.89 nN to 2.40 nN upon constraining the bond angles Fe–S1–C1 and Fe–S2–C2 to their equilibrium values. Bending the terminal bond angles therefore significantly facilitates the rupture of the Fe–S1 bond.

In addition, the structural anisotropy of the FeS_4_ unit is found to have an influence on the mechanochemistry of rubredoxin. The presence of the methyl groups in [Fe(iii)(SCH_3_)_4_]^−^ breaks the *T*_d_ symmetry of the FeS_4_ tetrahedron. While the Fe–S bond lengths are almost completely symmetric, the S–Fe–S bond angles in the investigated systems deviate by up to 6° from one another (*cf.* Tables S1 and S2†). As a result, the rupture forces of pulling different pairs of carbon atoms apart are different (Table S3†). In [Fe(iii)(SCH_3_)_4_]^−^ the rupture forces vary between 1.76 nN (coordinate C1–C4) and 1.93 nN (coordinate C1–C3). Similar effects are found when adding formamide molecules (Table S4†). These findings are in excellent agreement with the experimentally observed mechanical anisotropy of rubredoxin, substantiating further the quality of our results. Moreover, this shows the tremendous influence that minute changes in the internal coordinates, particularly bond angle bendings, have on the mechanical resilience of rubredoxin.

## 4 Conclusions and outlook

Using a combination of SMD and the quantum chemical JEDI strain analysis we showed that hydrogen bonds from neighboring amino acid backbones to the sulfur atoms of the FeS_4_ unit of rubredoxin become less prevalent when unfolding the protein and that they do not lower the rupture force, which excludes these hydrogen bonds as possible explanation of the low rupture force found in the experiment. Our protocol allowed us to track the distribution of strain energy during the mechanical unfolding of rubredoxin, providing a time-resolved view of the propagation of strain in the stretched protein in unprecedented detail. Using static strain analysis schemes, the structural anisotropy of the FeS_4_ pseudo-tetrahedron and angle bendings in this unit was shown to have an influence on the rupture properties of rubredoxin. Opening up the Fe–S–C bond angles leads to a decrease in rupture force, thereby sidestepping the necessity to rupture the Fe–S bond in an isolated manner. This is reminiscent of the concept of flex-activated mechanophores,^56,57^ in which angle bendings facilitate the rupture of a nearby covalent bond. However, due to the overestimation of the calculated rupture force compared to the experiments, it is clear that the model system used in the static quantum chemical calculations does not describe the experimental setup realistically and that the effects revealed by the static calculations are insufficient to explain the experimentally observed low rupture force of rubredoxin. Instead, other, previously unobserved mechanisms prevail, which will need to be investigated in detail using new experiments and computations. The novel combination of SMD and quantum chemical strain analysis used here will prove as a valuable tool for this purpose as well as for time-resolved studies of other classes of mechanically active proteins, thus paving the way for a detailed understanding of the propagation of strain through proteins during structural transitions.

## Conflicts of interest

There are no conflicts to declare.

## Supplementary Material

SC-011-D0SC02164D-s001
