# Supplementary material for: The rupture mechanism of rubredoxin is more complex than previously thought
Source: Chem Sci. 2020 May 27;11(23):6036–44. doi: 10.1039/d0sc02164d (PMC8159389; doi:10.1039/d0sc02164d)
Supplement: SC-011-D0SC02164D-s001 [file SC-011-D0SC02164D-s001.pdf]

Electronic Supplementary Material (ESI) for Chemical Science.
This journal is © The Royal Society of Chemistry 2020

Supporting Information

The Rupture Mechanism of Rubredoxin Is More Complex Than Previously Thought

Maximilian Scheurer,[1] Andreas Dreuw,[1] Martin Head-Gordon,[2,3] Tim Stauch[4,5,6]

[1] *Interdisciplinary Center for Scientific Computing, Im Neuenheimer Feld 205, 69120 Heidelberg, Germany*

[2] *Department of Chemistry, University of California, Berkeley, California 94720, United States of America*

[3] *Chemical Sciences Division, Lawrence Berkeley National Laboratory, University of California, Berkeley, California 94720, United States of America*

[4] *University of Bremen, Institute for Physical and Theoretical Chemistry, Leobener Straße NW2, D-28359 Bremen, Germany*

[5] *Bremen Center for Computational Materials Science, University of Bremen, Am Fallturm 1, D-28359 Bremen, Germany*

[6] *MAPEX Center for Materials and Processes, University of Bremen, Bibliothekstraße 1, D-28359 Bremen, Germany*

* Corresponding author. Email: tstauch@uni-bremen.de

Contents

1 **Benchmark of Density Functionals Against Experimental Data** 3

2 **Averaged Fe−S distances in SMD simulations** 8

3 **Localized Orbital Bonding Analysis** 9

4 **Rupture Force vs. Number of Hydrogen Bonds in [Fe(II)(SCH_3)$_4$]$^{2-}$** 11

5 **Fe−S Bond Lengths and Population Numbers in the Presence of Formamide Molecules** 12

6 **Structural Parameters of the [Fe(III/II)(SCH_3)$_4$]$^{-/2-}$ Model Systems** 14

7 **Rupture Forces Found in Different Pulling Coordinates** 15

8 **Detailed JEDI Analysis of [Fe(III)(SCH_3)$_4$]$^{-}$ and [Fe(II)(SCH_3)$_4$]$^{2-}$** 16

9 **CHELPG atomic charges for MD simulations** 17

1 Benchmark of Density Functionals Against Experimental Data

Various density functionals were benchmarked against experimental data. The tested density functionals comprise B3LYP,[1–3] B97,[4] B97M-rV,[5] BHHLYP,[1–3], BLYP,[1,2] BP86VWN,[1,6] M06-L,[7] PBE,[8] TPSS,[9] ωB97M-V,[10] and ωB97X-V.[11] Although multi-reference *ab initio* methods are of course to be preferred over Density Functional Theory (DFT) in the case of metal complexes, the large number of calculations needed to determine the rupture forces and the Hessian matrices for the JEDI analysis necessitate the use of a cost-efficient density functional. The def2-TZVP[12] basis set was used in all calculations for all atoms to provide a reliable description of the Fe−S bonds and the hydrogen bonds.

The performance of the density functionals was assessed by comparison to the experimental data provided by Day et al.,[13] who reported the bond lengths between Fe(III)/Fe(II) and the surrounding sulfur atoms in rubredoxin. Day et al. find that the bond lengths in the Fe(III) complex are shorter than in the Fe(II) complex, which agrees with chemical intuition. Also, those Fe−S bond lengths in which the sulfur atom is involved in two hydrogen bonds with neighboring amino acids are observed to be longer than if only one hydrogen bond is formed. Given that the protein environment has a significant influence on these structural parameters and only a small model system can be treated quantum chemically, it is unlikely that a single density functional can reproduce all experimental bond lengths. Nevertheless, it is desirable that the general trend is reproduced. Another crucial point is that the same density functional shall be used for all investigated systems so that the results for Fe(III) and Fe(II) can be compared to each other.

To model the protein environment more realistically, a total of six formamide molecule were added to the model systems $[Fe(III)(SCH_3)_4]^-$ and $[Fe(II)(SCH_3)_4]^{2-}$, which form hydrogen bonds to the sulfur atoms. As a result, two sulfur atoms are involved in two hydrogen bonds (mimicking Cys5 and Cys38) and the other two sulfur atoms are involved in only one hydrogen bond (mimicking Cys8 and Cys41).

The results of the benchmark are given in Figures S1 and S2. As expected, the performance of the density functionals is very diverse and no single functional reproduces all experimental parameters correctly. Particularly in the case of Cys8 and Cys41, in which one hydrogen bond is involved, all tested functionals overestimate the experimentally observed Fe−S bond length. Nevertheless, most functionals reproduce the general trend that those Fe−S bonds in which the sulfur atom is involved in two hydrogen bonds are longer than in those cases in which only one hydrogen bond is formed. We found that the BP86VWN functional offers an attractive compromise between agreement with the experiment and computational cost, so we chose this functional for calculations reported in the paper. Moreover, BP86VWN

was used successfully before to reproduce experimental Fe−S bond lengths in model complexes.[14] It is noted in passing that the application of a solvent model for water (C-PCM,[15,16] $\epsilon = 78$) generally leads to a deterioration of the agreement with the experimental data (Figures S3 and S4), so we ran all our calculations in the gas phase.

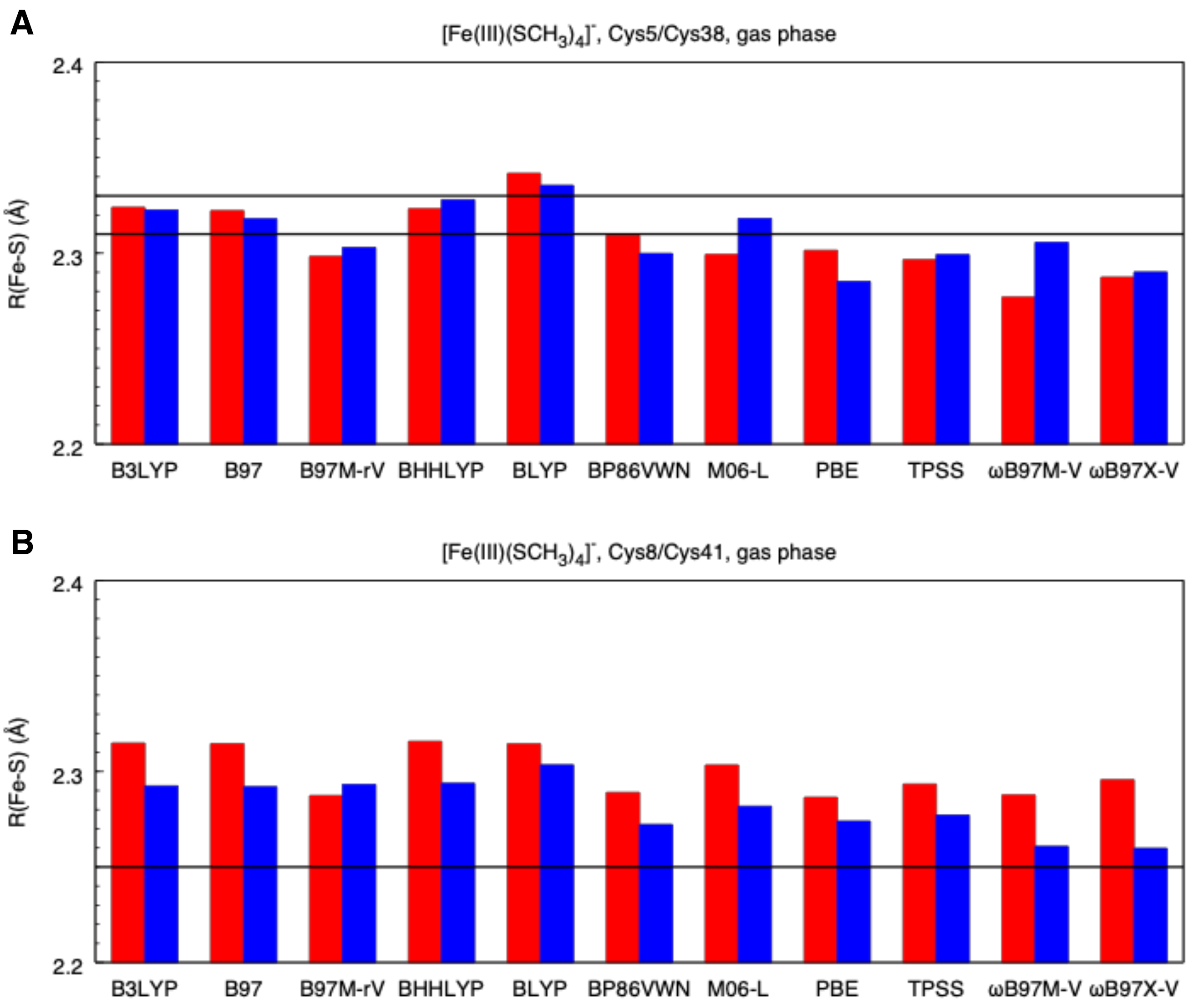

Figure S1: Distances between the Fe(III) atom and the S atoms of Cys5 and Cys38 (A) as well as Cys8 and Cys41 (B), calculated with various density functionals in the gas phase for the $[Fe(III)(SCH_3)_4]^-$ model system with six hydrogen bonds. The horizontal lines represent the experimental values of 2.31 Å (Cys5), 2.33 Å (Cys38) and 2.25 Å (Cys 8 and Cys41) reported by Day et al.[13]

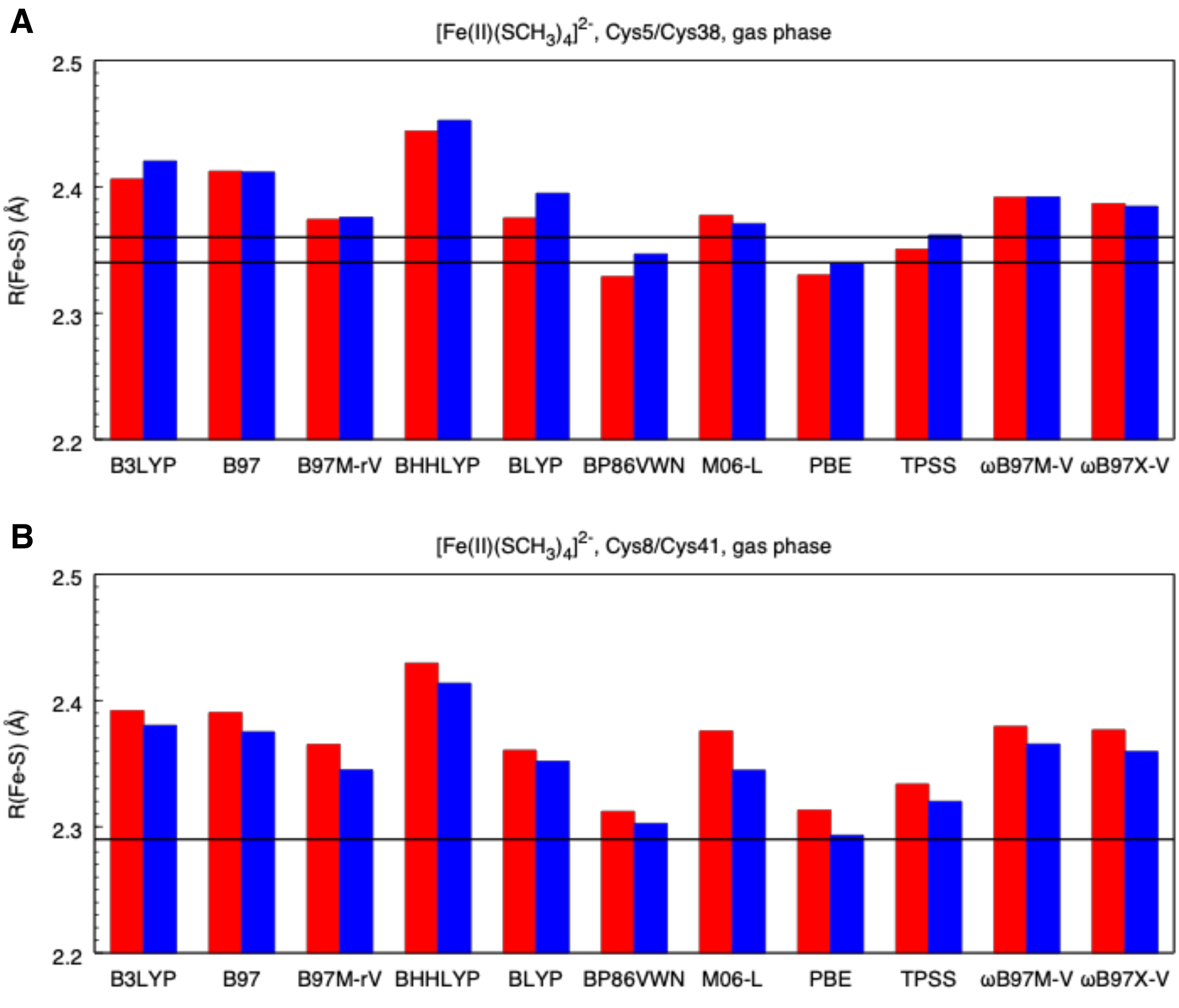

Figure S2: Distances between the Fe(II) atom and the S atoms of Cys5 and Cys38 (A) as well as Cys8 and Cys41 (B), calculated with various density functionals in the gas phase for the $[Fe(II)(SCH_3)_4]^{2-}$ model system with six hydrogen bonds. The horizontal lines represent the experimental values of 2.34 Å (Cys5), 2.36 Å (Cys38) and 2.29 Å (Cys 8 and Cys41) reported by Day et al.[13]

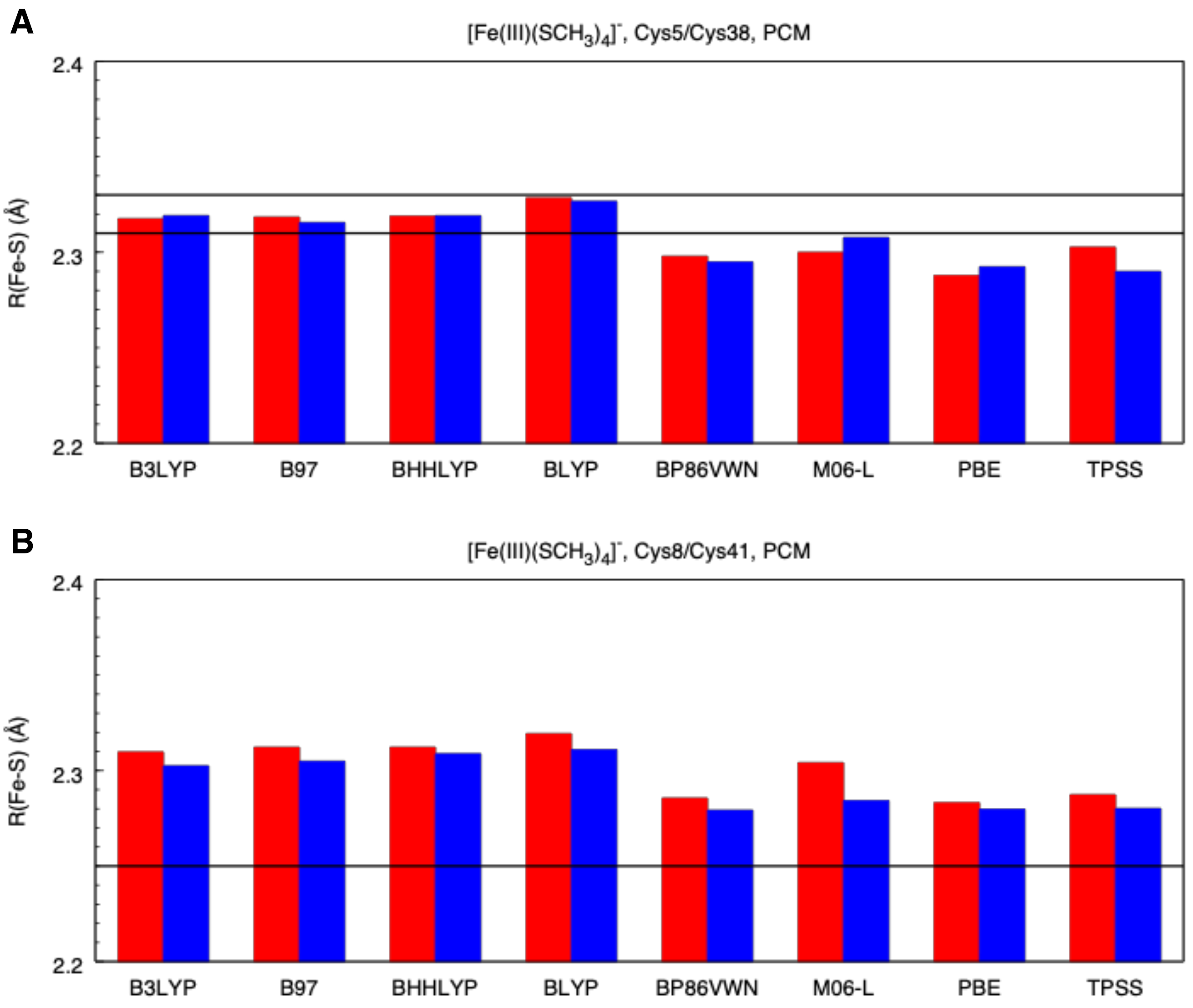

Figure S3: Distances between the Fe(III) atom and the S atoms of Cys5 and Cys38 (A) as well as Cys8 and Cys41 (B), calculated with various density functionals using the Polarizable Continuum Model (PCM, solvent: water, $\epsilon = 78$) for the $[Fe(III)(SCH_3)_4]^-$ model system with six hydrogen bonds. The horizontal lines represent the experimental values of 2.31 Å (Cys5), 2.33 Å (Cys38) and 2.25 Å (Cys 8 and Cys41) reported by Day et al.[13]

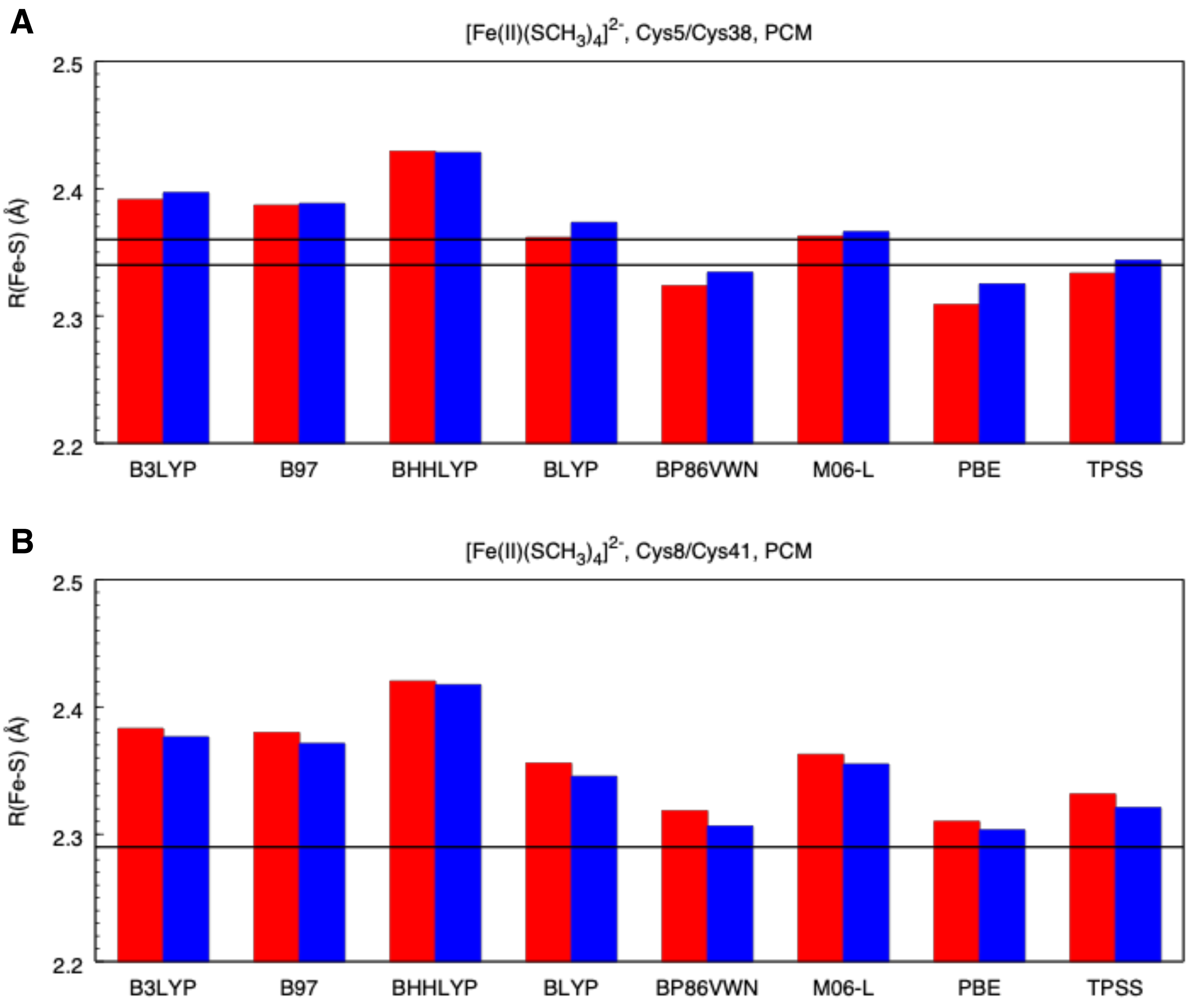

Figure S4: Distances between the Fe(II) atom and the S atoms of Cys5 and Cys38 (A) as well as Cys8 and Cys41 (B), calculated with various density functionals using the Polarizable Continuum Model (PCM, solvent: water, $\epsilon = 78$) for the $[Fe(II)(SCH_3)_4]^{2-}$ model system with six hydrogen bonds. The horizontal lines represent the experimental values of 2.34 Å (Cys5), 2.36 Å (Cys38) and 2.29 Å (Cys 8 and Cys41) reported by Day et al.[13]

2 Averaged Fe−S distances in SMD simulations

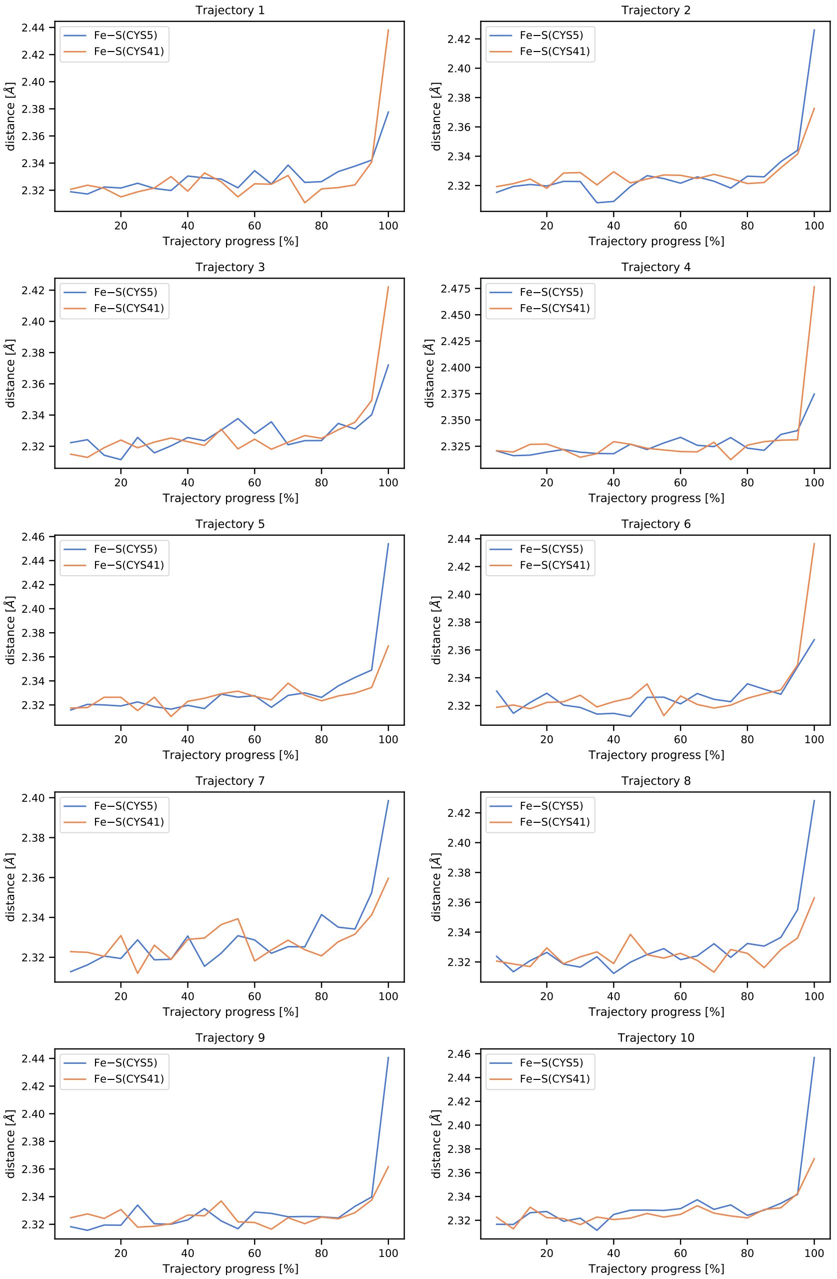

Figure S5: Fe−S distances averaged for 20 equally sized trajectory windows of all ten SMD trajectories. At 100% trajectory progress, the first bond ruptures.

3 Localized Orbital Bonding Analysis

The Localized Orbital Bonding Analysis (LOBA) provides an in-depth view of the bonding situation in the model complexes. In general, similar Löwdin population numbers on two atoms involved in a bond are interpreted as a sign for covalency. However, in typical heteronuclear bonds a disparity in the occupation numbers is found, the degree of which has been suggested to be a measure of covalent bond strength.[17] In $[Fe(III)(SCH_3)_4]^-$, each of the four sulfur atoms forms a bond with the central iron atom, as evidenced by the Edmiston-Ruedenberg (ER) orbitals in combination with the Löwdin population numbers (Figure S6). These bonds are formed in the space of β-electrons by a donation of one of the three lone pairs of the sulfur atoms in the SCH_3^- ligands into the partially filled d-orbitals of iron. The Löwdin occupation numbers on the iron and sulfur atoms are 0.33 and 0.62, respectively, emphasizing that the electron density is not shared equally between iron and sulfur but instead donated by the sulfur atoms. Nevertheless, the covalent character of the Fe(III)−S bonds in $[Fe(III)(SCH_3)_4]^-$ is distinctly higher than in $[Fe(II)(SCH_3)_4]^{2-}$, where the disparity between the population numbers on the iron and sulfur atoms is more pronounced (Fe(II): 0.27, S: 0.68, Figure S7), which at least partially explains the higher rupture force found in $[Fe(III)(SCH_3)_4]^-$.

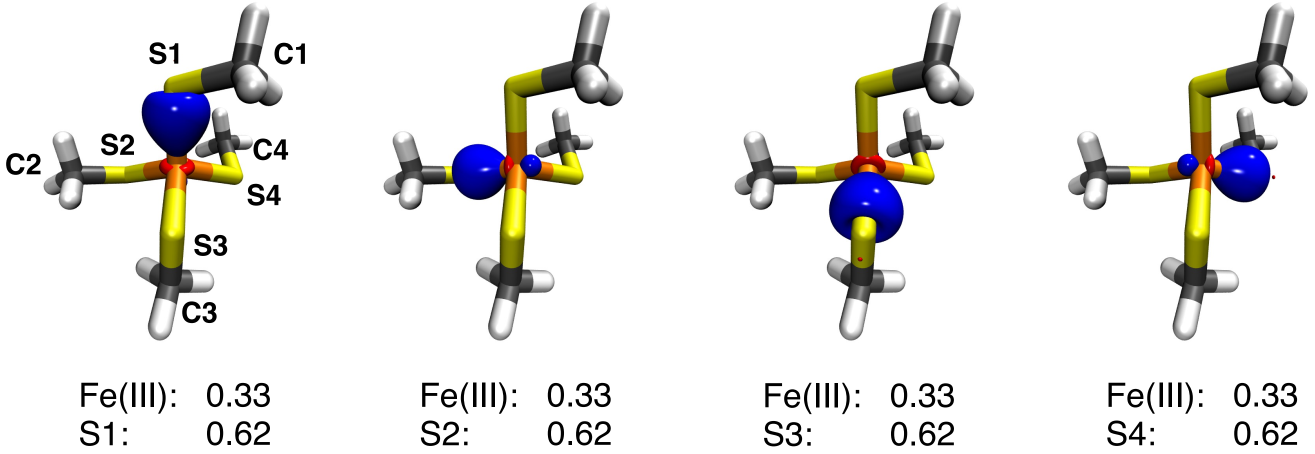

Figure S6: Isosurfaces (isovalue = 0.10 a.u.) and Löwdin population numbers of the ER orbitals (β-spins) of the Fe(III)−S bonds in $[Fe(III)(SCH_3)_4]^-$. The plots were created using VMD.[18]

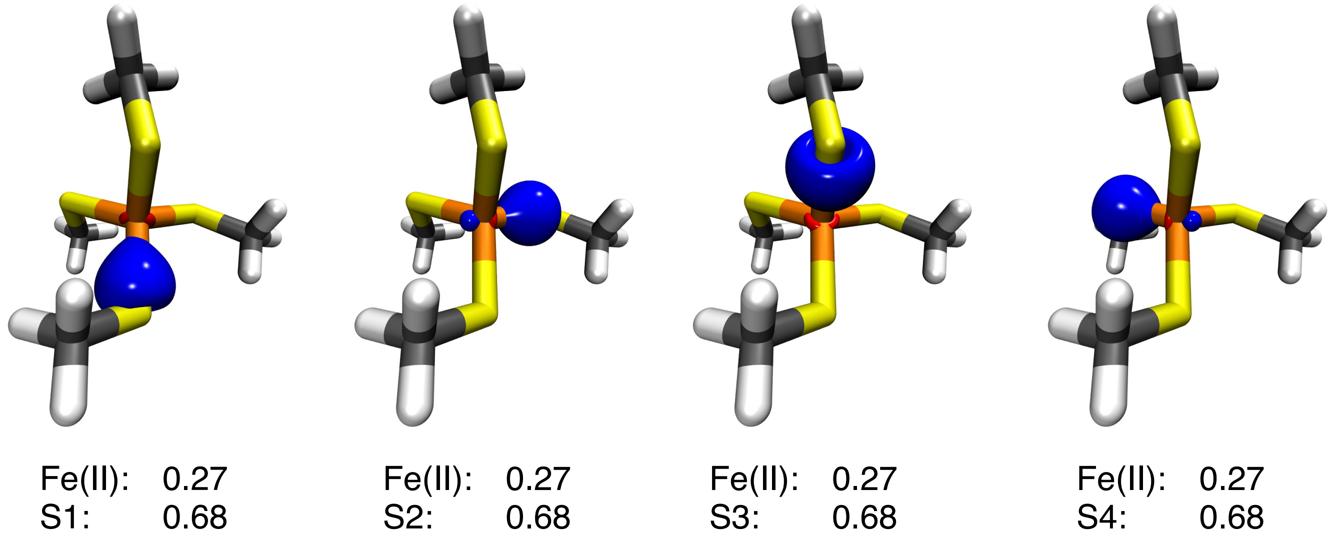

Figure S7: Isosurfaces (isovalue = 0.10 a.u.) and Löwdin population numbers of the ER orbitals (β-spins) of the Fe(II)−S bonds in $[Fe(II)(SCH_3)_4]^{2-}$. The plots were created using VMD.[18]

4 Rupture Force vs. Number of Hydrogen Bonds in $[Fe(II)(SCH_3)_4]^{2-}$

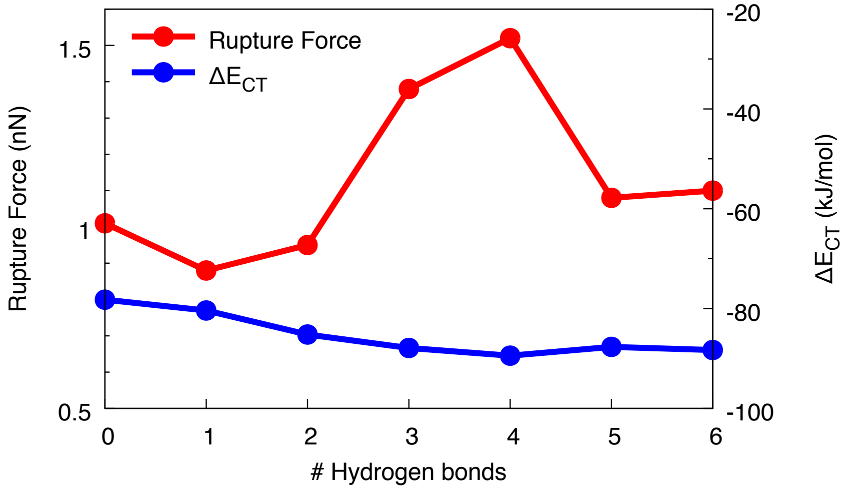

Figure S8: Rupture forces and charge transfer energies ΔE_{CT} for $[Fe(II)(SCH_3)_4]^{2-}$ where a varying number of hydrogen atoms is formed between the sulfur atoms and formamide molecules. Lines were included to guide the eye.

In contrast to $[Fe(III)(SCH_3)_4]^{-}$, the changes in charge-transfer energy in $[Fe(II)(SCH_3)_4]^{2-}$ when more and more hydrogen bonds are added are relatively small and ΔE_{CT} decreases almost monotonically upon addition of formamide molecules. As evidenced by the progression of the Löwdin population numbers of the ER orbitals of the Fe(II)−S bonds when adding formamide molecules (cf. Figure S10), this can indeed be linked to a slight increase in covalent character of the Fe(II)−S bond. However, since the covalent character of the Fe(II)−S bond is less pronounced than in the case of Fe(III)−S, the importance of the charge-transfer term for the bonding interaction between Fe(II) and S is much lower.

5 Fe−S Bond Lengths and Population Numbers in the Presence of Formamide Molecules

The Fe−S bond lengths in the $[Fe(III)(SCH_3)_4]^-$ and $[Fe(II)(SCH_3)_4]^{2-}$ model systems as a function of the number of hydrogen bonds formed between formamide molecules and the sulfur atoms are given in Figure S9. Although the Fe−S bond length can increase significantly when the sulfur atom is involved in a hydrogen bond with formamide, the Löwdin population numbers of the ER orbitals remain close to their initial values (Figure S10) during the addition process. Except for an outlier in $[Fe(III)(SCH_3)_4]^-$ with three formamide molecules, the occupation numbers even change slightly into the direction of increasing covalency. This demonstrates that the covalent character of the model system is not perfectly correlated with the number of hydrogen bonds, so that the low rupture force in the experiment cannot be attributed to this effect.

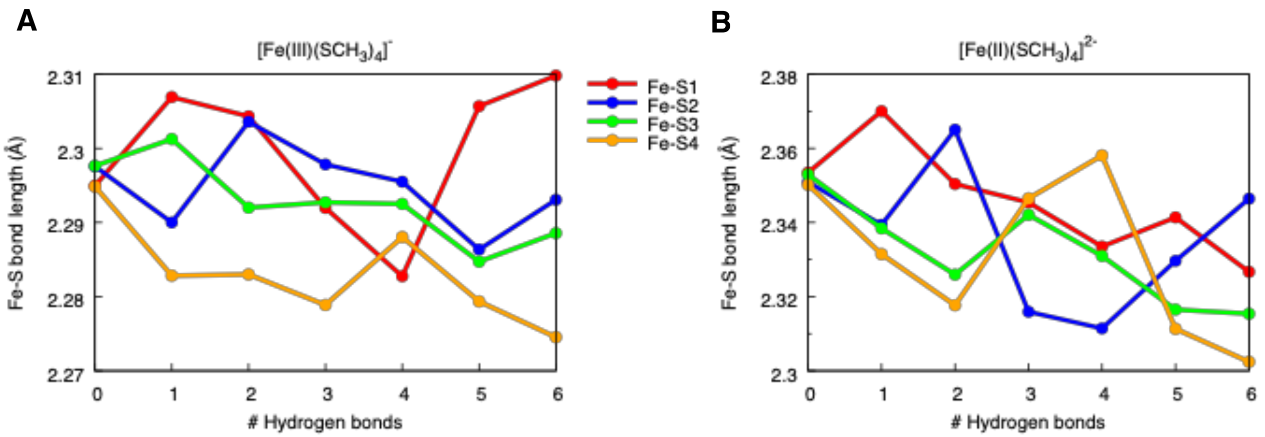

Figure S9: Fe−S bond lengths of the model systems $[Fe(III)(SCH_3)_4]^-$ (A) and $[Fe(II)(SCH_3)_4]^{2-}$ (B) upon addition of formamide molecules that form hydrogen bonds with the sulfur atoms. Formamide molecules were added to the sulfur atoms in the order S1, S2, S3, S4, S1, and S2. Lines were included to guide the eye.

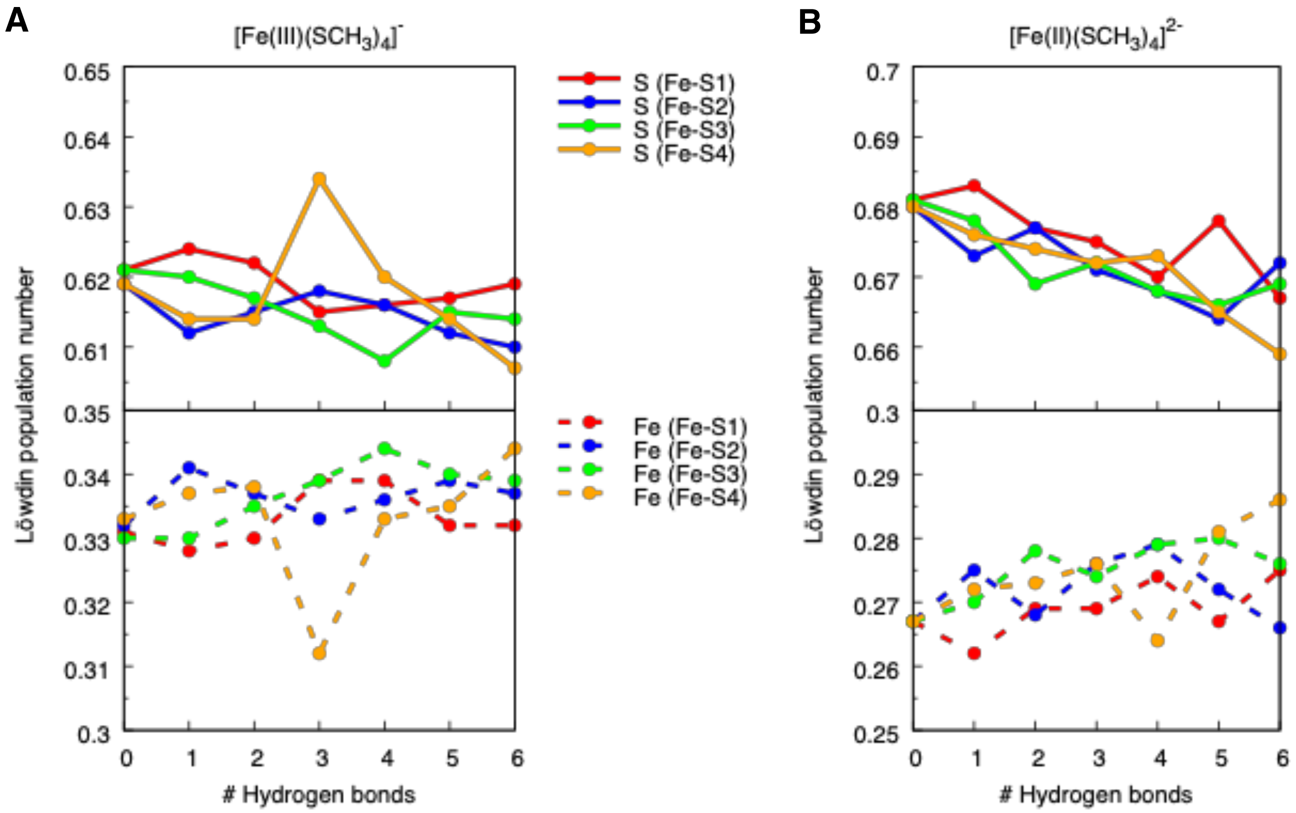

Figure S10: Löwdin population numbers of the ER orbitals of the Fe−S bonds in $[Fe(III)(SCH_3)_4]^-$ (A) and $[Fe(II)(SCH_3)_4]^{2-}$ (B) upon addition of formamide molecules that form hydrogen bonds to the sulfur atoms. The population numbers on the sulfur atom are shown in the top panel (solid lines), whereas those on the iron atom are shown in the bottom panel (dashed lines). Formamide molecules were added to the sulfur atoms in the order S1, S2, S3, S4, S1, and S2. Lines were included to guide the eye.

6 Structural Parameters of the $[Fe(III/II)(SCH_3)_4]^{-/2-}$ Model Systems

Structural parameters (Fe−S bond lengths and S−Fe−S bond angles) in $[Fe(III)(SCH_3)_4]^-$ and $[Fe(II)(SCH_3)_4]^{2-}$ with no hydrogen bonds are given in Tables S1 and S2. The numbering scheme is the same as in the main paper.

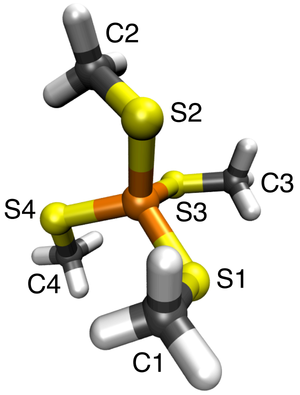

Bond length	$[Fe(III)(SCH_3)_4]^-$ (Å)	$[Fe(II)(SCH_3)_4]^{2-}$ (Å)
Fe−S1	2.30	2.35
Fe−S2	2.30	2.35
Fe−S3	2.30	2.35
Fe−S4	2.30	2.35

Table S1: Fe−S bond lengths in $[Fe(III)(SCH_3)_4]^-$ and $[Fe(II)(SCH_3)_4]^{2-}$ at the BP86VWN/def2-TZVP level of theory.

Bond angle	$[Fe(III)(SCH_3)_4]^-$ (deg)	$[Fe(II)(SCH_3)_4]^{2-}$ (deg)
S1−Fe−S2	109.8	107.1
S1−Fe−S3	108.9	107.8
S1−Fe−S4	109.6	113.3
S2−Fe−S3	109.0	113.4
S2−Fe−S4	108.5	107.3
S3−Fe−S4	111.0	108.2

Table S2: S−Fe−S bond angles in $[Fe(III)(SCH_3)_4]^-$ and $[Fe(II)(SCH_3)_4]^{2-}$ at the BP86VWN/def2-TZVP level of theory.

7 Rupture Forces Found in Different Pulling Coordinates

Pulling coordinate	$F_{rup}([Fe(III)(SCH_3)_4]^-)$ (nN)	$F_{rup}([Fe(II)(SCH_3)_4]^{2-})$ (nN)
C1−C2	1.89	1.01
C1−C3	1.93	1.00
C1−C4	1.76	1.13
C2−C3	1.77	1.12
C2−C4	1.87	1.00
C3−C4	1.83	1.00

Table S3: Rupture forces F_{rup} determined for different pulling coordinates in $[Fe(III)(SCH_3)_4]^-$ and $[Fe(II)(SCH_3)_4]^{2-}$. The numbering scheme is the same as in the main text.

# H-bonds S1,S2	$F_{rup}([Fe(III)(SCH_3)_4]^- \cdot (HCONH_2)_6)$ (nN)
2−2	1.79
2−1	1.68
1−1	1.97

Table S4: Rupture forces F_{rup} in $[Fe(III)(SCH_3)_4]^- \cdot (HCONH_2)_6$ when pulling apart the carbon atoms attached to sulfur atoms that are involved in a varying number of hydrogen bonds. In 2−1, for example, one of the sulfur atoms is involved in two hydrogen bonds and the other sulfur atom is involved in only one hydrogen bond.

8 Detailed JEDI Analysis of $[Fe(III)(SCH_3)_4]^-$ and $[Fe(II)(SCH_3)_4]^{2-}$

The mechanical anisotropy of the $[Fe(III)(SCH_3)_4]^-$ pseudo-tetrahedron and the importance of the bendings for the rupture process can be observed in the progression of the harmonic strain energies stored in the different internal coordinates with increasing stretching force (Figure S11A). The bond angle bendings Fe−S1−C1 and Fe−S2−C2 store most strain energy throughout the entire stretching coordinate, signifying that they are softer than the central bending S1−Fe−S2. Hence, in the static strain analysis, the bond angles in the FeS_4 unit store more strain energy than in the dynamic calculations, signifying that different points of view on the rupture process of rubredoxin are provided by the two approaches. The strain in the Fe−S bonds strongly increases with increasing force. This effect is more pronounced for the Fe−S1 bond as a result of the observed anisotropy and preconditions this bond for rupture. Not surprisingly, the C−S bonds are stronger and do not store as much strain energy as the Fe−S bonds. Qualitatively similar results were obtained for $[Fe(II)(SCH_3)_4]^{2-}$ (Figure S11B).

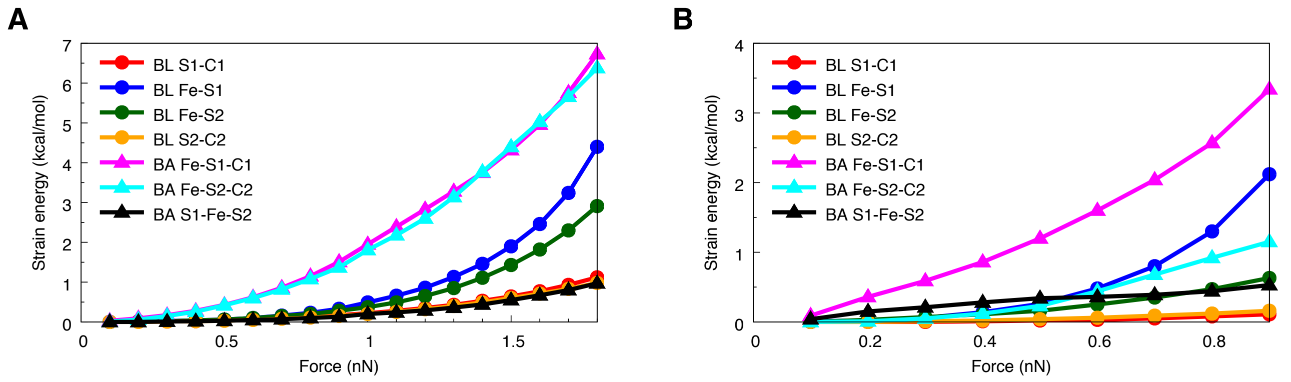

Figure S11: Harmonic strain energies stored in various bond lengths (BL) and bond angles (BA), calculated with the JEDI analysis for $[Fe(III)(SCH_3)_4]^-$ (A) and for $[Fe(II)(SCH_3)_4]^{2-}$ (B). Due to the large amount of torsions in the molecule the discussion is restricted to the mechanically most relevant bonds and bendings. The numbering scheme is the same as in the main paper.

9 CHELPG atomic charges for MD simulations

The atomic charges of the rubredoxin active site, comprising four deprotonated cysteine residues and the Fe(III) ion were re-parametrized by means of quantum-chemical calculations. The procedure included a geometry optimization of the minimal model system (see main text), representing the side chains of the respective amino acids, at the PBE[8]/6-31G*[19] level of theory as implemented in the ORCA program package.[20] Afterwards, CHELPG charges[21] were calculated and assigned to the corresponding CHARMM atom types for subsequent MD simulations. The used parameters are summarized in Table S5.

Table S5: Charges used in MD simulations

residue name	atom name	charge [e]
FE3P	FE3P	0.823381
CYM	SG	-0.531905
CYM	CB	-0.011612
CYM	HB1	0.0439395
CYM	HB2	0.0439395

References

[1] A. D. Becke, *Phys. Rev. A*, 1988, **38**, 3098–3100.

[2] C. Lee, W. Yang and R. G. Parr, *Phys. Rev. B*, 1988, **37**, 785–789.

[3] A. D. Becke, *J. Chem. Phys.*, 1993, **98**, 1372–1377.

[4] A. D. Becke, *J. Chem. Phys.*, 1997, **107**, 8554–8560.

[5] N. Mardirossian, L. Ruiz Pestana, J. C. Womack, C. K. Skylaris, T. Head-Gordon and M. Head-Gordon, *J. Phys. Chem. Lett.*, 2017, **8**, 35–40.

[6] J. P. Perdew, *Phys. Rev. B*, 1986, **33**, 8822–8824.

[7] Y. Zhao and D. G. Truhlar, *J. Chem. Phys.*, 2006, **125**, 194101.

[8] J. P. Perdew, K. Burke and M. Ernzerhof, *Phys. Rev. Lett.*, 1996, **77**, 3865–3868.

[9] J. Tao, J. P. Perdew, V. N. Staroverov and G. E. Scuseria, *Phys. Rev. Lett.*, 2003, **91**, 146401.

[10] N. Mardirossian and M. Head-Gordon, *J. Chem. Phys.*, 2016, **144**, 214110.

[11] N. Mardirossian and M. Head-Gordon, *Phys. Chem. Chem. Phys.*, 2014, **16**, 9904–9924.

[12] F. Weigend, M. Häser, H. Patzelt and R. Ahlrichs, *Chem. Phys. Lett.*, 1998, **294**, 143–152.

[13] M. W. Day, B. T. Hsu, L. Joshua-Tor, J.-B. Park, Z. H. Zhou, M. W. W. Adams and D. C. Rees, *Protein Sci.*, 1992, **1**, 1494–1507.

[14] A. Dey, T. A. Okamura, N. Ueyama, B. Hedman, K. O. Hodgson and E. I. Solomon, *J. Am. Chem. Soc.*, 2005, **127**, 12046–12053.

[15] V. Barone and M. Cossi, *J. Phys. Chem. A*, 1998, **102**, 1995–2001.

[16] M. Cossi, N. Rega, G. Scalmani and V. Barone, *J. Comput. Chem.*, 2003, **24**, 669–681.

[17] A. J. W. Thom, E. J. Sundstrom and M. Head-Gordon, *Phys. Chem. Chem. Phys.*, 2009, **11**, 11297–11304.

[18] W. Humphrey, A. Dalke and K. Schulten, *J. Mol. Graphics*, 1996, **14**, 33–38.

[19] W. J. Hehre, R. Ditchfield and J. A. Pople, *J. Chem. Phys.*, 1972, **56**, 2257–2261.

[20] F. Neese, *WIREs Comput. Mol. Sci.*, 2012, **2**, 73–78.

[21] C. M. Breneman and K. B. Wiberg, *J. Comput. Chem.*, 1990, **11**, 361–373.
